# Biochip Technology for the Serological Diagnosis of Bullous Pemphigoid

**DOI:** 10.5402/2012/237802

**Published:** 2012-12-17

**Authors:** Haik Zarian, Andrea Saponeri, Anna Michelotto, Edoardo Zattra, Anna Belloni-Fortina, Mauro Alaibac

**Affiliations:** Unit of Dermatology, University of Padua, Via Battisti 206, 35121 Padua, Italy

## Abstract

Bullous pemphigoid is an autoimmune blistering skin disease characterized by the presence of circulating autoantibodies which recognize specific proteins of the epidermis and dermoepidermal junction. Diagnosis is based on clinical criteria and laboratory investigations, notably histology, direct and indirect immunofluorescence, and ELISA. This study describes a new immunofluorescence assay for parallel determination of anti-BP180 and anti-BP230 based on recombinant antigenic substrates. The aim of the study was to detect BP180 and BP230 autoantibodies by BIOCHIP technology using both a specially designed recombinant BP180-NC16A protein and cells expressing the BP230-gc antigen fragment. 18 patients with bullous pemphigoid were included in the study. Autoantibodies to BP180 were detected by the BIOCHIP technique in 83.33% of patients with clinical, serological, and immunohistological confirmed bullous pemphigoid while autoantibodies against BP230-gC were detected only in 39% of patients. The detection of anti-BP180-NC16A and anti-BP230-gC by a new biochip-based immunoassay is a suitable alternative to indirect immunofluorescence and ELISA. This method has the advantage of easily discriminating the different autoantibody specificities. The BIOCHIP method is faster, cheaper, and easy to use when compared with the ELISA approach. For this reason, the new method could be used as an initial screening test to identify patients with bullous pemphigoid, and doubtful results could then be confirmed by ELISA.

## 1. Introduction

Bullous pemphigoid (BP) is an autoimmune blistering skin disease characterized by subepidermal bullae and circulating autoantibodies directed against the basement membrane zone of stratified squamous epithelia [1]. Several studies have confirmed that these autoantibodies react predominantly with two main components of hemidesmosomes, bullous pemphigoid antigen 1 (BPAG1, also termed BP230), and bullous pemphigoid antigen 2 (BPAG2, also BP180 or type XVII collagen) [2]. Hemidesmosomes are specialized multiprotein complexes that mediate epithelial-stromal cohesion in stratified and complex epithelia and connect the intermediate filament system of basal epithelial cells to proteins of the extracellular matrix. These junctional complexes are formed by at least six different types of proteins: laminin-332 (laminin-5), the integrin  *α*6*β*4, the bullous pemphigoid antigens 180 (BP180, BPAG2, or type XVII collagen) and 230 (BP230 or BPAG1-e), CD151, and plectin [3–5].

BP is characterized by the presence of antibodies primarily targeting two structural hemidesmosomal proteins: BP180 and BP230. BP180 is a transmembrane glycoprotein of about 1500 amino acids which plays an important role in mediating anchorage between the cell surface at the site of the hemidesmosome. The extracellular portion of the 16th noncollageneous (NC16A) was identified as the immunodominant region of BP180 in patients with BP [6]. BP230 is an intracellular protein of the hemidesmosomal component belonging to the plakin family of cytolinkers. The immunodominant epitopes are preferentially localized in the globular C-terminal domain of BP230 that mediates interaction with keratin filaments [7, 8]. The vast majority of BP patients possess anti-BP180 autoantibodies, and this parameter plays a crucial role in diagnosis of this autoimmune blistering disease, while the determination of anti-BP230 antibodies may support the serological analysis by an additional parameter in selected cases [9]. 

Direct immunofluorescence (DIF) and serological classic indirect immunofluorescence (IIF) using human salt-split skin sections and/or monkey oesophagus sections have been for long time the main tests utilised to diagnose autoimmune blistering conditions. Here, we describe a new IIF assay for parallel determination of anti-BP180 and anti-BP230, based on recombinant antigenic substrates [10]. This innovative immunoassay provides a useful alternative to immunoassays based on purified antigens, such as ELISA and immunoblot. Autoantibodies against BP180 are identified with extremely high efficiency using a specially designed recombinant protein encompassing the NC16A portion of the extracellular domain of BP180. The recombinant protein is coated directly onto BIOCHIPs. For the detection of autoantibodies against BP230 cells expressing the BP230-gC antigen fragment are utilised.

## 2. Material and Methods

The study comprised 18 caucasian patients with PB, 12 females, age ranging from 47 to 90, and 6 males, age ranging from 29 to 84 (Table 1). Serum samples from 3 normal healthy individuals and 2 patients with pemphigus vulgaris were used as negative controls.

The diagnosis of BP was made on the basis of clinic, histologic, and immunopathologic criteria. In particular, the specific circulating antibodies were detected by enzyme immunoassay ELISA, utilizing a recombinant form of the NCA16 domain of BP180 (MBL, Nagoya, Japan). The cut-off value was 9 U/mL. Recombinant DSG1 and DSG3 proteins were used as further control (MBL, Nagoya, Japan). The cut-off values were 20 U/mL and 14 U/mL, respectively. 

**Figure 1:**
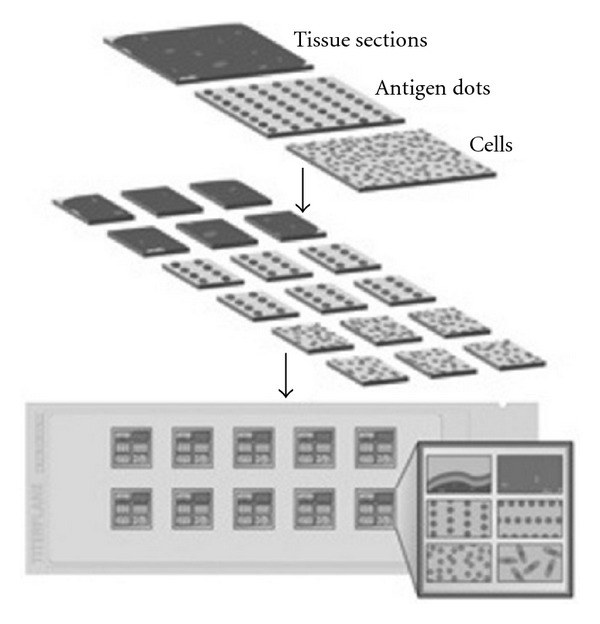
BIOCHIP mosaic.

**Figure 2:**
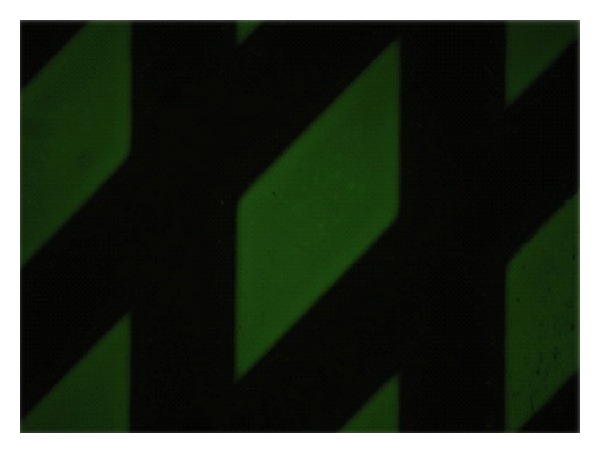
Positive reaction to BP180 using recombinant tetrameric NC16A spots.

**Figure 3:**
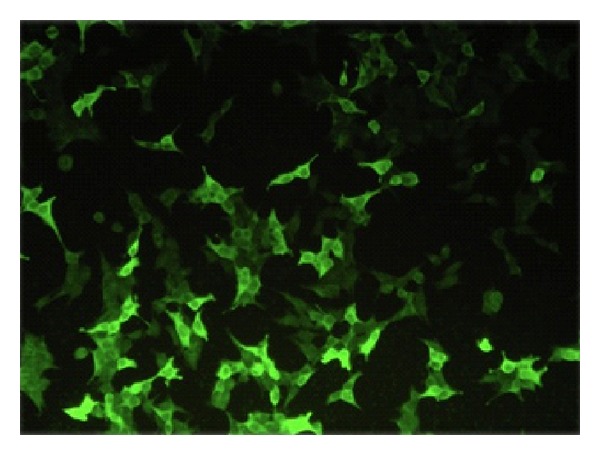
Positive reaction to BP230 using cells expressing the BP230-cG fragment.

For the detection of autoantibodies by BIOCHIP Technology [10], we utilised the recombinant tetrameric BP180-NC16A spotted on coverglasses and further fragmented on “BIOCHIPs.” Autoantibodies against BP230 were detected using cells expressing the BP230-gC fragment, characterized by a higher sensitivity than the full-length protein (BP230FL). In our study the BIOCHIP mosaic consisted of 2 substrates: BP230-gC-expressing cells and recombinant tetrameric BP180-NC16A spots (*Euroimmun Italia, Padova*, http://www.euroimmun.it/). The BIOCHIP Mosaic can be customised with further substrates if required, notably human salt-split skin sections, monkey oesophagus sections, desmoglein-1-expressing cells, and desmoglein-3-expressing cells. This test is designed exclusively for the in vitro determination of human antibodies in serum. Combinations of different substrates are tested with diluted patient sample. If a positive reaction is obtained, specific antibodies attach to the antigens. In a second step the attached antibodies are stained with fluorescein-labelled anti-human antibodies and made visible with a conventional fluorescence microscope. For the production of BIOCHIP slides a method similar to that is used by the electronics industry in the manufacture of microchip. In this approach, the substrates are no longer applied directly to microscope slides, but initially to thin glass slides. These are mechanically cut into millimetre-sized fragments (BIOCHIPs). The BIOCHIPs are then glued onto microscope slides using automated assembly equipment (Figure 1). With this method it is possible to produce large batches of each substrate, thus reducing fluctuations in quality. The miniature size of the BIOCHIPs means that the reaction fields of the slides can be supplemented with further BIOCHIP substrates if required (BIOCHIP Mosaic). In the first incubation step (30 minutes) the substrates are incubated with diluted (1/10) patient serum samples. In the second incubation step (30 minutes) the attached antibodies are stained with fluorescein-labelled anti-human antibodies. Results are evaluated visually by conventional fluorescence microscopy (Figures 2 and 3).

## 3. Results

Autoantibodies to BP180 were detected in 83.33% of patients (15/18) with BP. There were no positive results in the negative control group. False negative results were observed in 16% (3/18) of samples and correlated with the presence of low autoantibodies titre as measured by ELISA (Tables 1 and 2). Autoantibodies to anti-BP230-gC were detected in 39% (7/18) of patients with BP (Tables 1 and 2). There were no positive results in the control group. All patients without detectable autoantibodies to BP180 showed a negative result against BP230-gC. 

## 4. Discussion

The aim of the study was to evaluate the diagnostic value and performance of a new IIF test to detect antibodies against BP180 and BP230 in relation to the ELISA method. Specifically, ELISA has many advantages: it is a minimally invasive technique, it allows performing the analysis of multiple samples simultaneously and in a limited time, it is an easily reproducible method, and it provides a quantitative analysis [11]. A limitation of the ELISA method is that the recombinant proteins used may not contain all of the epitopes present in vivo. A final disadvantage is the high cost of the method. 

The BIOCHIP technique has proved to be a specific and sensitive diagnostic alternative to the ELISA diagnosis of BP [10, 12]. A BIOCHIP mosaic, prepared using different tissue sections, cell substrates or antigenic molecules, requires only a simple antibody incubation to obtain a detailed profile, so it is possible to search simultaneously different antibodies directed against different organs or infectious agents. Some of the advantages of the BIOCHIP technology include the possibility to analyse simultaneously several serum samples, the short time necessary for performing test (about 100 minutes), the easy interpretation of results which are interpreted visually and does not require spectrophotometric equipment, making it possible also in small laboratories. Furthermore all incubation steps proceed at room temperature and there is low reagent consumption: only 50 *μ*L each of diluted serum and reagent are needed per test field. Finally, unlike ELISA, in which we have specific kits for a single parameter, with this new IIF based test, there may be more parameters in a single session using only one kit. Therefore the BIOCHIP method is fairly cheap compared to the ELISA methods although we have not yet made a detailed cost analysis. The main disadvantage of BIOCHIP technique against ELISA is the fact that it does not provide a quantitative value. 

Although our study was based on a relatively small group of patients, our results were comparable with previous investigations [10, 12]. Taken together, these cumulative findings indicate that, in the determination of autoantibodies to BP180, the diagnostic specificity of the BIOCHIP method was almost comparable to the ELISA [10, 12], whereas in the detection of anti-BP230-gC the diagnostic sensibility for BP230 was slightly reduced [10, 12]. These data indicate that BIOCHIP method may replace the classical IIF, which can be considered outdated in some ways. Using several BIOCHIPs coated with different substrates side by side on one and the same reaction field, antibodies against different skin target structures can be investigated simultaneously, notably BP180, BP230, DSG1, and DSG3. Therefore, this new immunoassay can be used as an excellent screening test for patients with suspected autoimmune bullous skin disease, preserving the more expensive ELISA test in doubtful cases. 

In conclusion, the novel BIOCHIP described here is a flexible, specific, and sensitive alternative to detect autoantibodies by IIF and ELISA in the diagnostic workup of BP. This new method can not only be applied to the diagnosis and screening of BP, but also enrich the serological diagnosis of other autoimmune diseases by additional parameters. The BIOCHIP technology proved to be a valuable complementary diagnostic tool, for the simplicity of execution, low cost, and high sensitivity. 

## Figures and Tables

**Table 1:** Results at the time of diagnosis. ELISA values are expressed as Unit/mL. Biochip results are expressed as positive or negative.

Patient no.	Age	sex	Disease	DSG1	DSG3	BP180	BP180	BP230
				(ELISA)	(ELISA)	(ELISA)	(BIOCHIP)	(BIOCHIP)
1	82	F	Pemphigoid	—	—	160.34	+	−
2	77	F	Pemphigoid	3.25	—	72.38	+	−
3	58	F	Pemphigoid	—	1.47	153.12	+	+
4	80	F	Pemphigoid	1.13	1.02	88.91	+	+
5	92	F	Pemphigoid	3.5	1.12	155.54	+	+
6	49	F	Pemphigoid	9.25	5.31	136.64	+	+
7	64	F	Pemphigoid	—	—	80.38	+	−
8	76	F	Pemphigoid	—	—	66.58	+	−
9	88	F	Pemphigoid	0	0	156.33	+	−
10	82	F	Pemphigoid	1.48	0	76.84	+	+
11	77	M	Control	0	0	0	−	−
12	51	M	Pemphigoid	0.9	0.7	195.3	+	−
13	61	M	Pemphigoid	0	0	38.27	+	−
14	86	F	Control	0	0	1.68	−	−
15	92	F	Pemphigoid	4.6	0	86.06	+	+
16	86	M	Pemphigoid	0	0	177.5	+	+
17	74	F	Pemphigoid	0	0	19.90	−	−
18	48	M	Control	—	—	6.67	−	−
19	86	M	Pemphigoid	—	—	51.17	+	−
20	29	M	Pemphigoid	—	—	11.05	−	−
21	48	F	Pemphigus	12.88	116.51	—	−	−
22	46	M	Pemphigus	44.45	70.13	0.2	−	−
23	23	F	Pemphigoid	—	—	33.01	−	−

**Table 2:** Summary of results in BP patients.

Autoantibody	Number of positive cases	Percentage of positive cases
ANTI-BP180-NC16a	15/18	83.33
ANTI-BP230-gC	7/18	39%
